# Incidence of Incisional Hernia after Cesarean Delivery: A Register-Based Cohort Study

**DOI:** 10.1371/journal.pone.0108829

**Published:** 2014-09-30

**Authors:** Anna J. M. Aabakke, Lone Krebs, Steen Ladelund, Niels J. Secher

**Affiliations:** 1 Department of Obstetrics and Gynecology, University of Copenhagen, Holbæk Hospital, Holbæk, Denmark; 2 Clinical Research Center, University of Copenhagen, Hvidovre Hospital, Hvidovre, Denmark; 3 The Research Unit Women’s and Children’s Health, The Juliane Marie Center, Copenhagen University Hospital, Copenhagen, Denmark; 4 Department of Obstetrics and Gynecology, Aarhus University Hospital, Aarhus, Denmark; Oslo University Hospital, Ullevål, Norway

## Abstract

**Objective:**

To estimate the incidence of incisional hernias requiring surgical repair after cesarean delivery over a 10-year period.

**Methods:**

This population- and register-based cohort study identified all women in Denmark with no history of previous abdominal surgery who had a cesarean delivery between 1991 and 2000. The cohort was followed from their first until 10 years after their last cesarean delivery within the inclusion period or until the first of the following events: hernia repair, death, emigration, abdominal surgery, or cesarean delivery after the inclusion period. For women who had a hernia repair, hospital records regarding the surgery and previous cesarean deliveries were tracked and manually analyzed to validate the relationship between hernia repair and cesarean delivery. Data were analyzed with a competing risk analysis that included each cesarean delivery.

**Results:**

We identified 57,564 women who had had 68,271 cesarean deliveries during the inclusion period. During follow-up, 134 of these women had a hernia requiring repair. Of these 68 (51% [95% CI 42–60%]) were in a midline incision although the transverse incision was the primary approach at cesarean delivery during the inclusion period. The cumulated incidence of a hernia repair within 10 years after a cesarean delivery was 0.197% (95% CI 0.164–0.234%). The risk of a hernia repair was higher during the first 3 years after a cesarean delivery, with an incidence after 3 years of 0.157% (95% CI 0.127–0.187%).

**Conclusions:**

The overall risk of an incisional hernia requiring surgical repair within 10 years after a cesarean delivery was 2 per 1000 deliveries in a population in which the transverse incision was the primary approach at cesarean delivery.

## Introduction

Cesarean delivery is the most common surgical procedure in the United States, and the rates are increasing worldwide [1], [2]. In the United States, Latin America, Australia, and several European countries cesarean delivery rates are presently above 30% [3]–[6]. Several studies have found both short- and long-term complications related to cesarean delivery [7]–[11]. Incisional hernia is a well-known long-term complication of abdominal surgery, with a reported incidence of 3.0–20.6% in association with midline incisions and 0–2.1% with lower transverse incisions [12]–[19]. Two meta-analyses have confirmed that the risk of developing an incisional hernia after abdominal surgery is higher after a midline than a transverse incision [20], [21]. However, only one study, with 6 to 12 months follow-up, has focused on incisional hernia after cesarean delivery [12]. The authors found no incisional hernias after 280 cesarean deliveries performed through a transverse incision. Studies have found that more than 50% of incisional hernias occur more than 1 year after surgery, indicating that the incidence of incisional hernia after cesarean delivery might be underreported [16], [22].

The objective of this study was to estimate the incidence of incisional hernias requiring surgical repair after cesarean delivery in a large cohort during a 10-year period.

## Materials and Methods

This was a population-based cohort study with data obtained from the Danish National Patient Register and the Danish Medical Birth Register.

In Denmark all in- and out-patient hospital contacts are registered in the Danish National Patient Register established in 1977 and all births in the Danish Medical Birth Register established in 1968 [23], [24]. Both registers were administered by the Danish National Board of Health at the time of data extraction. All Danish citizens have a unique personal identification number, and information in the registers is linked to this number. The registers contain information on age, gender, surgical procedure, diagnosis, readmission, and reoperation, but no surgical details (e.g. type of incision and suture material). Surgical procedures have since 1996 been recorded based on codes according to the NOMESCO classification of surgical procedures (a common Nordic classification) [25]. Before 1996, surgical registrations were based on a national classification of treatments and surgical procedures. The completeness of the registration in the Danish National Patient Register is 98–100%, and the validity of the system in terms of the type of surgical procedure has been proven to be high [26]–[28].

This study was based on a search in the two registries. The main inclusion criterion was a cesarean delivery between January 1, 1991 and December 31, 2000 in women with no history of previous abdominal surgery. Women who had had a cesarean delivery during the inclusion period were identified through the codes for cesarean delivery (sectio_u and, sectio_f [1991–95], and KMCA 10, 11, 12a, 12b [1996–2000]). The cohort was followed from their first cesarean delivery until 10 years after their last cesarean delivery in the inclusion period for an event of hernia repair. Hernia repairs performed during 1991–95 were identified by the codes herniotomia incisionalis (40680), herniotomia ventralis (42400), herniotomia parietis abdominis (40600), hernioplastica cum implantation (40800), hernioplastica cum implantatione laparascopica (40801), and herniotomia (40740, 42900, and no previous diagnosis of inguinal hernia). Hernia repairs during 1996–2000 were identified by the code for surgeries of incisional hernia (KJAD). We registered only the primary hernia repair, and any recurrences were not included. We also registered events of death, emigration, abdominal surgery, and cesarean deliveries during the follow-up period. The surgical codes for hernia repair do not contain information about the anatomical location of the hernia. Therefore, for women with a hernia repair, hospital records of the primary examination, description of surgery, and discharge letter were retrieved as were the descriptions of the previous cesarean deliveries. The records were manually analyzed by two investigators (AA and LK) to validate the relationship between hernia repair and cesarean delivery and to determine the type of incision. When in doubt, consensus was obtained through discussion. Before study initiation, the following exclusion criteria for the hernia repairs were defined: Diastasis recti without hernia, hernia not in the cesarean incision, and no hernia. Cases in which no validation information was accessible were maintained in the cohort as confirmed cases so that the incidence of hernia repair was not falsely underestimated.

### Ethics Statement

The results of the study were reported following the STROBE recommendations for strengthening the reporting of observational studies in epidemiology [29].

The study was approved by the Danish Data Protection Agency (reg. no. 2013-41-2155). Ethical approval was not required. Written informed consent was not required and therefore not obtained for the clinical records to be used in the study. However, patient records and the data-set were anonymized before analysis.

### Statistics

The cumulative risk of a hernia repair after cesarean delivery was estimated by a competing risk analysis [30]. Each cesarean delivery during the inclusion period was analyzed individually. The competing endpoints that mutually excluded each other were hernia repair, death, abdominal surgery, and a consecutive cesarean delivery. Emigration during the follow-up period caused censoring. Right censoring also occurred after 10 years if the woman did not meet any of the endpoints mentioned above.

Continuous data were analyzed by unpaired *t* test when normally distributed and by Mann Whitney test when not. Nominal data were analyzed by chi^2^ test or, when relevant, Fischer’s exact test. *P* values <0.05 were regarded as statistically significant.

The analyses were performed using R 3.0.2 (R Foundation for Statistical Computing, Vienna, Austria) with the add-on library cmprsk [30], and IBM SPSS Statistics 21 (SPSS Inc., Chicago, IL, USA).

## Results

**Figure 1:**
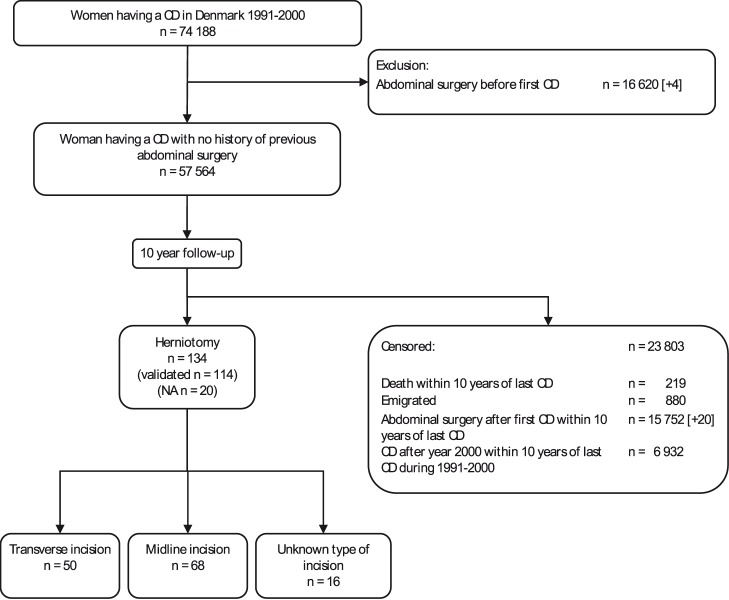
Flow through the study. Number in brackets is number of excluded and censored cases found through validation. CD: cesarean delivery. NA: Not available.

Between January 1991 and December 2000, 57,564 unique women, with no history of previous abdominal surgery, had a total of 68,271 cesarean deliveries. Of these, 158 were identified as having a subsequent hernia repair performed within 10 years after their cesarean delivery within the inclusion period. Analysis of the hospital records excluded 24 cases. Of these, 4 women had a history of previous abdominal surgery, 4 had abdominal surgery after the cesarean delivery but before the hernia repair, one had the hernia repair done coincidently with the including cesarean delivery, 9 hernias were not in the cesarean incision, 5 had diastasis recti without hernia, and 1 had no hernia. A total of 134 cases of hernia repair were included in the analysis. In 20 patients, hospital records from the hernia repair surgery were not available, primarily due to destruction of medical records after 10 years of inactivity. In 68 cases (51% [95% CI 42–59%]) the hernias treated were in a midline incision, in 50 cases (37% [95% CI 29–46%]) in a transverse incision, and in 16 cases (12% [95% CI 7–19%]) in an incision of unknown type due to missing cesarean delivery records. Study design and flow including information on competing events are illustrated in Figure 1. The basic characteristics of the hernia repairs based on type of incision did not differ and are shown in Table 1. There was no change over time in either the frequency of cesarean deliveries resulting in hernia repairs or the distribution of transverse and midline incisions among the hernias requiring hernia repair (data not shown).

**Table 1:** Basic characteristics of surgical repairs performed on hernias in midline and transverse incisions.

	Transverse incision	Midline incision	*P*
	(n = 50)	(n = 68)	
Age at last cesarean delivery before hernia repair (years) [Mean (SD)]	32 (5)	32 (5)	0.836^*^
Number of cesarean deliveries before hernia repair [Median (IQR)]	1.5 (1.0–2.0)	2.0 (1.0–2.8)	0.139^†^
Time from cesarean delivery to hernia repair (months) [Median (IQR)]	18.5 (9.6–26.0)	21.7 (11.4–60.8)	0.078^†^
Acute hernia repair [n (%)]	2 (4.5%)	2 (3.1%)	1.000^‡^

Data analyzed with *unpaired *t*-test, ^†^Mann Whitney test, and ^‡^ Fischer’s exact test.

**Figure 2:**
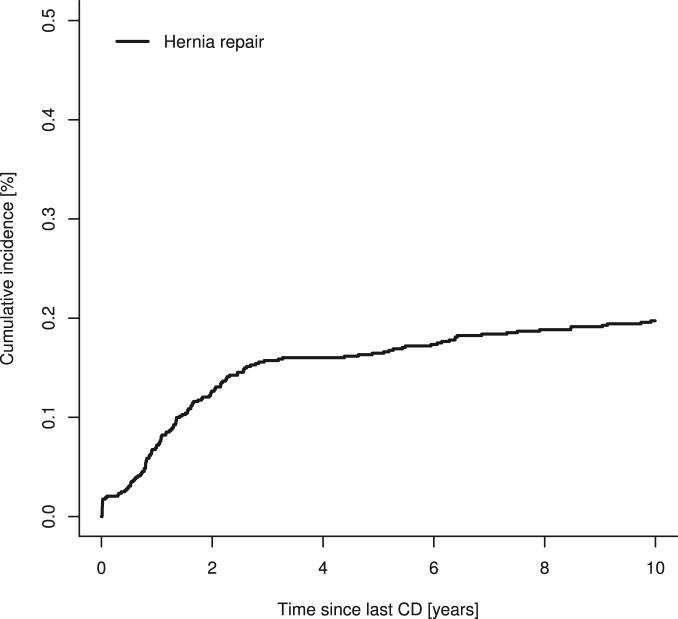
Cumulative incidence of incisional hernias requiring repair after cesarean delivery. CD: cesarean delivery.

The cumulated incidence of a hernia repair within 10 years after a cesarean delivery was 0.197% (95% CI 0.164–0.234%) (Figure 2). The risk of a hernia repair was higher within the first 3 years after a cesarean delivery, with a cumulated incidence at 3 years of 0.157% (95% CI 0.127–0.187%) (Figure 2 and Table 2). Among women who developed an incisional hernia requiring surgical repair the median time from cesarean delivery to repair was 1.36 years (IQR 0.79–2.27 years).

**Table 2:** Cumulative incidence of incisional hernias requiring surgical repair 1, 3, and 10 years after a cesarean delivery.

	1 year	3 years	10 years
Hernia repair	0.072 (0.052–0.092)	0.157 (0.127–0.187)	0.197 (0.164–0.231)

Data are expressed as % (95% confidence interval).

## Discussion

This prospective cohort study found a cumulated risk of 0.197% of developing an incisional hernia requiring surgical repair within 10 years after a cesarean delivery. The risk of a hernia repair was higher during the first 3 years after a cesarean delivery.

Less than half of the hernia repairs were performed within the first year after the cesarean delivery (Table 2). Our study thereby confirms previous studies showing that less than half of incisional hernias develop within the first year after surgery [22]. However, it cannot be ruled out that women who developed an incisional hernia within the first year after a cesarean delivery postponed repair of various reasons.

Through validation, we found that the repairs identified in this study were of hernias in both midline and transverse incisions and that more than half of them were of hernias in midline incisions. During the study period, the majority of cesarean deliveries in Denmark were performed through a transverse incision, but, unfortunately, the distribution of the two types of incisions during the study period is unknown. We therefore can only try to make an estimate of the risk of surgical repair of a hernia in transverse incisions based on previously published data: In order not to underestimate the risk, unknown incision types are assumed to be transverse and the following assumptions regarding midline incisions are made. The lowest found incidence of incisional hernia in midline incisions is reported to be 3.0% in gynecological surgeries [13]. Additionally, one study found that 16% of incisional hernias occurring within 10 years of laparotomy are surgically repaired [16]. Based on these findings, surgical hernia repair was performed on 0.48% of all midline incisions in the study population. Under these assumptions surgical repair of transverse incision hernias was performed after 66 of 54.104 cesarean deliveries during a 10 year period corresponding to an overall crude incidence of 0.12%. The risk of a hernia in a transverse incision requiring repair is therefore probably lower than the risk found in this study. Previous studies have found that the risk of developing a hernia in a midline compared with a transverse incision is increased, with odds ratios of 1.68 to 3.33 [20], [21]. This difference most likely explains why more than half the hernia repairs in our study were performed on hernias in midline incisions, although the percentage of midline incisions in the cesarean delivery cohort was low. We found no statistically significant differences between the hernia repairs performed in a midline compared with transverse incision regarding time from cesarean delivery to repair, number of previous cesarean deliveries, age at the cesarean delivery, or percentage performed as an emergency procedure.

Factors other than the type of incision have been suggested to influence the development of incisional hernias. The suture material and suture technique used to close the fascia have been shown to affect the risk of incisional hernia in midline incisions. A suture technique with continuous sutures placed 1 cm apart and 1 cm from the incision using a suture 4 times the length of the incision has been shown to prevent hernias [31]. Additionally, a meta-analysis found that slowly absorbable suture material caused fewer hernias [32]. Unfortunately, there are no studies on closure techniques of the fascia in transverse incisions for cesarean delivery. The risk of incisional hernia after cesarean delivery seems to be so low that randomized trials with incisional hernia as the primary outcome would need a very large number of participants and at least 3 years of follow-up. And unfortunately, two current multifactorial randomized trials with long-term follow-up after cesarean delivery are not exploring methods to close the fascia [33], [34]. The development of incisional hernias may also be influenced by factors such as BMI and post-cesarean complications including infection. Unfortunately these variables were not available in the Danish registers during the study period and were not uniformly registered in the available medical records, and could therefore not be included in this analysis. A case-control study could theoretically evaluate differences in various risk factors between cases with a hernia repair and a control group. However, since data about BMI, post-operative infection, and surgical technique for fascial closure were not consistently registered in the records in the 1990s, the study would only be able to add information about risk differences due to incision type and confirm previously estimated odds ratios.

This is the largest study to date that gives an estimate of the risk of developing an incisional hernia requiring repair after cesarean delivery and the first study with a follow-up time of more than 1 year. However, the study has some limitations that need to be considered. Register studies are dependent on the rate, completeness, and accuracy of the registration. Both the registration rate and the validity of type of surgical procedures in the Danish National Patient Register have been shown to be high, and we assume that our data are representative [26]–[28]. The surgical codes for hernia repair do not contain information about anatomical location of the hernia explaining the relatively high rate of hernia repairs that were found to be unrelated to a previous cesarean delivery through validation. We excluded women with previous abdominal surgery from our cohort, and there may have been some cases of hernia repairs after a cesarean delivery in this group. However, it is unlikely that the incidence is different in the excluded group compared with our cohort, and thus exclusion of these women should not have affected our results.

The Danish National Patient Register was established in 1977, and patients with abdominal surgeries performed before 1977 could not be identified and excluded from our cohort. Additionally, we excluded some cases of hernia repair in the process of validation due to the identification of previous abdominal surgeries. This may have caused us to underestimate the actual incidence of hernia repair after cesarean delivery. But there are also some factors that may have led to an overestimation of the actual incidence of hernia repair after cesarean delivery. During the process of validation, a number of hospital records were not retrievable. We maintained these unconfirmed cases in the cohort in order to avoid underestimation. Additionally, more than half the repairs were of hernias in midline rather than transverse incisions, the latter being the preferred incision for cesarean delivery in the Western world [35]. Unfortunately, the distribution of the two types of incisions in Denmark in the 1990s is unknown, although the transverse incision was the incision of choice. We were therefore not able to separately estimate the risk of a hernia repair in the two types of incisions.

The risk of developing an incisional hernia after a cesarean delivery that does not require repair was not investigated in this study. The validity of diagnostic codes in the Danish registries have been shown to be poor [36], [37] and we therefore selected the surgical code of hernia repair. Medical treatment is free in Denmark and we therefore assume that clinically important incisional hernias were treated surgically due to the risk of serious adverse events (e.g. incarceration). But small hernias may be left un-treated, and thus the risk of an incisional hernia might be higher than the risk of surgical hernia repair found in this study.

In conclusion, this study found that the overall risk of having an incisional hernia requiring repair within 10 years of a cesarean delivery was 2 per 1000 cesarean deliveries. Most hernias were in midline incisions in a population in which the transverse incision was the primary approach at cesarean delivery. Consequently the risk of developing a hernia in a transverse cesarean incision following current surgical recommendations is very low and should not restrict the use of cesarean delivery.
